# 核苷转运蛋白基因*ENT3*单核苷酸多态性与肺癌易感性的关系

**DOI:** 10.3779/j.issn.1009-3419.2010.05.15

**Published:** 2010-05-20

**Authors:** 雪飞 李, 颉 张, 增利 张, 彩存 周

**Affiliations:** 1 200433 上海，同济大学医学院附属上海市肺科医院肺癌免疫研究室 Laboratory of Lung Cancer and Immunology, Shanghai Pulmonary Hospital, Affiliated to Tongji University School of Medicine, Shanghai 200433, China; 2 200433 上海，同济大学医学院附属上海市肺科医院肿瘤科 Department of Medical Oncology, Shanghai Pulmonary Hospital, Affiliated to Tongji University School of Medicine, Shanghai 200433, China

**Keywords:** 核苷转运蛋白, 单核苷酸多态性, 探针, 肺肿瘤, 遗传易感性, Equilibrative nucleoside transporter, Single nucleotide polymorphism, Probe, Lung neoplasms, Hereditary susceptibility

## Abstract

**背景与目的:**

核苷转运蛋白介导的核苷跨膜转运在调节细胞功能中发挥重要作用，可能是某些肿瘤的候选易感基因。本研究旨在探讨核苷转运蛋白基因*ENT3*单核苷酸多态性与肺癌易感性的关系。

**方法:**

采用病例对照研究，收集2008年5月-2009年5月于上海市肺科医院就诊的原发性肺癌患者351例和同期住院的非肿瘤患者207例，应用AllGlo^TM^探针结合实时荧光PCR方法分析肺癌组和对照组*ENT3*基因rs10999776多态位点的基因型分布情况，比较不同基因型与肺癌易感性的关系以及不同基因型联合吸烟对肺癌易感性的影响。肺癌组与对照组基因型和等位基因分布比较用χ^2^检验，以调整比值比及95%CI表示相对危险度，所有统计检验均为双侧概率检验，所有资料均用SPSS软件进行统计。

**结果:**

肺癌患者rs10999776多态位点的CC、TC、TT基因型和C、T等位基因频率分布与对照组比较无统计学差异（*P* > 0.05）。与非吸烟的野生型纯合子个体比较，携带突变等位基因T（TC+TT）的吸烟个体罹患肺癌的风险性明显增加，且吸烟≥30包/年者风险性更高，调整OR值分别为2.848（95%CI: 1.536-4.879, *P*=0.005）、3.076（95%CI: 2.308-6.741, *P*=0.001）。而对肺癌不同组织类型的分析发现，三种基因型的吸烟个体罹患肺鳞癌的风险性均增加，且携带突变等位基因T的个体风险性更高，调整OR值为6.066（95%CI: 2.884-12.758, *P* < 0.001）。而rs10999776（C＞T）多态性联合吸烟对肺腺癌则无显著影响。

**结论:**

核苷转运蛋白基因*ENT3* rs10999776多态性可能与肺鳞癌的发病风险相关，且与吸烟环境暴露存在一定的相互作用。

## Abstract (en)

**Background and objective:**

The transport of nucleoside transmembrane mediated by equilibrative nucleoside transporter (ENT) plays an important role in regulating various cellular functions, and the *ENT* gene may be candidate gene of tumors. The aim of this study is to investigate the association between the single nucleotide polymorphism (SNP) of ENT3 and the hereditary susceptibility of lung cancer.

**Methods:**

A case-control study was performed involved in 351 lung cancer patients and 207 cancer-free controls from Chinese population in Shanghai pulmonary hospital. The rs10999776 (C > T) polymorphism was determined by using Real-time PCR with AllGlo^TM^ probes. The frequency distribution of genotypes and allele between lung cancer and controls groups was analyzed by *chi*-*square* test. The association between polymorphism in the *ENT3* gene with the risk of lung cancer was estimated by computing odds ration (OR) and 95%CI.

**Results:**

The genotype (CC, TC, TT) and allele distribution of the ENT3 SNP in the patients with lung cancer was not significantly different compared with that in controls (*P* > 0.05). Compared with never-smokers with wild homozygous genotype, smokers with T allele (TC+TT) had increased risk of lung cancer (OR=2.848, 95%CI: 1.536-4.879, *P*=0.005), and those with pack-years of smoking more than 30 had higher risk (OR=3.076, 95%CI: 2.308-6.741, *P*=0.001). And the risk of squamous cell carcinoma significantly increased in smokers, especially those with T allele (TC+TT) genotype (OR=6.066, 95%CI: 2.884-12.758, *P* < 0.001). The genotype with smoking conditions had no significant effect on adenocarcinoma (all *P* > 0.05).

**Conclusion:**

The results suggested rs10999776 polymorphism may implicate in the risk of squamous cell carcinoma in Chinese population which may interact with smoking-exposure.

过去20年间，中国肺癌的发病率和死亡率均在显著增加。研究^[1]^表明吸烟是肺癌的主要危险因素之一。然而，只有一部分暴露个体会罹患肺癌，这说明个体的遗传背景可能与肺癌的易感性有关。目前已发现很多基因如DNA修复基因、原癌基因、细胞周期调节基因等，它们的遗传变异或单核苷酸多态性均与肺癌易感性相关。

平衡型核苷转运蛋白是一类膜内在蛋白，根据浓度梯度完成核苷及其类似物的跨膜转运。目前该家族有4个成员：ENT1-4。核苷作为体内重要的低分子化合物具有很多重要功能。除了参与RNA和DNA的合成之外，它们还参与细胞代谢、细胞信号转导等诸多重要生理功能^[2]^。而由核苷转运蛋白所介导的核苷跨膜转运在调节细胞功能中发挥重要作用，同时对于核苷酸的从头合成也是极其重要的。另外核苷转运蛋白也是核苷类似物抗肿瘤或抗病毒药物吸收的关键因素，可能影响药物疗效。已有文献^[3]^报道，这一家族成员SLC29A1即*ENT1*是结直肠癌的候选易感基因。另外，Yamamoto等^[4]^报道ENT3多态位点rs10999776（C＞T）与晚期非小细胞肺癌（non small cell lung cancer, NSCLC）一线化疗后的总体生存时间具有相关性。然而，*ENT3*基因的单核苷酸多态性（single nucleotide polymorphism, SNP）是否与肺癌易感性具有相关性尚未见报道。该研究通过病例-对照研究，探讨中国汉族人群ENT3多态位点rs10999776（C＞T）与肺癌易感性的关系。

## 1 材料与方法

### 1.1 材料

351例肺癌患者为2008年5月-2009年5月在上海市肺科医院诊断并经病理确诊的原发性肺癌初诊患者，未经过任何抗癌治疗，无职业性致癌因素接触史，均为汉族人群。病理类型按WHO《肺肿瘤组织学分型》（2000）标准进行分类，其中腺癌192例，鳞癌100例，混合癌31例（腺鳞混合癌、腺癌大细胞混合癌、鳞癌小细胞混合癌、低分化癌），未分类28例。207例对照为上海市肺科医院同期住院的非肿瘤患者。研究个体之间无血缘关系。每天至少吸1支，连续吸烟1年或以上者定义为吸烟个体。包/年=（每日吸烟支数÷20支）×吸烟年数。

### 1.2 DNA提取和资料收集

所有研究对象均采集外周静脉血5 mL，应用外周血DNA抽提试剂盒（上海超世生物）抽提基因组DNA，具体方法按说明书操作。经Elution buffer溶解的基因组DNA置于-20 ℃冰箱保存备用。所有研究个体均签署了知情同意书。

### 1.3 ENT3 rs10999776多态性检测

#### 1.3.1 引物及探针合成

引物及探针均由上海市超世生物技术有限公司合成。序列如下：上游引物5’-GCCAAGAACAAAACAGTGAGC-3’，下游引物5’-CAAGGAATGGCCAGGAGAA-3’；AllGlo^TM^野生型探针MARTTCCTACCCCGGAGAGA-MAR，突变型探针MARTCTTCTCTCCAGGGT-MAR。AllGlo^TM^探针为新一代荧光探针（专利号：60/23263），两端均标记MAR荧光基团，两者互为报告基团和淬灭基团，大大提高了荧光增量，同时因为染料上含有可以提高Tm值的基团，也使探针的特异性大大提高。

#### 1.3.2 实时荧光PCR反应体系及反应条件

反应体系为20 μL，其中Mix buffer 10 μL，上下游引物各250 nmol/L，模板DNA 100μL，Taq-DNA聚合酶1 U。每个样品分别在2个毛细管内进行反应，每管分别含有250 nmol/L的野生型探针和突变型探针。反应条件：95 ℃预变性15 s，95 ℃、10 s，58 ℃、40 s，45个循环。实时荧光PCR仪Lightcycler 3.0购自德国罗氏公司。

#### 1.3.3 结果判断

实时PCR产物为85 bp。探针利用与靶序列特异性杂交以显示扩增产物的增加。在探针与靶序列配对时，两端的荧光基团互为淬灭基团无荧光信号发出，当延伸进行时，DNA聚合酶5’外切酶活性使得2个荧光基团分离而同为报告基团，从而检测到荧光信号。每个样本在2个分别含有野生型和突变型探针的毛细管内进行反应，若在含有野生型探针的毛细管内发生了延伸反应，检测到呈S形曲线的荧光信号，而在另一毛细管内无反应，则该样本为野生型纯合子（CC）；反之，则为突变型纯合子（TT）。若两管均发生延伸反应，产生S形曲线，则样本为杂合子（TC）。然后随机抽取个别样本进行DNA测序检测，以验证探针反应的准确性，测序工作由上海超世生物技术有限公司完成。

### 1.4 统计学分析

所有资料应用SPSS 13.0进行分析。采用χ^2^检验分析对照组rs10999776多态位点基因型分布的*Hardy*-*Weinberg*平衡。肺癌组和对照组的基因型及等位基因分布比较采用χ^2^检验。应用非条件*Logistic*回归模型计算相对风险度的比值比（odds ratio, OR）及95%CI，以*P*＜0.05为差异具有统计学意义。

## 2 结果和分析

### 2.1 肺癌组与对照组的基本情况

如表 1所示，肺癌组和对照组的性别和年龄构成无统计学差异（*P*＞0.05）。肺癌组吸烟个体比例（56.1%）显著高于对照组（46.4%）（χ^2^=4.962, *P*=0.026）。累积吸烟量（包/年）肺癌组与对照组比较也具有统计学差异（χ^2^=10.692, *P*=0.001）。

**1:** 肺癌组和对照组一般情况与吸烟情况比较 The comparison of general condition and smoking situation between lung cancer and control group

Variables	Lung cancer (*n*=351)			Control (*n*=207)		*P*
	*n*	Constituent ratio (%)		*n*	Constituent ratio (%)	
Age						0.249
< 60	162	46.2		106	51.2	
> 60	189	53.8		101	48.8	
Gender						0.106
Male	239	68.1		127	61.4	
Female	112	31.9		80	38.6	
Smoking status						0.026
Non-smokers	154	43.9		111	53.6	
Smokers	197	56.1		96	46.4	
Cumulative smoking						0.001
< 30	58	29.4		47	49.0	
> 30	139	70.6		49	51.0	

### 2.2 ENT3 rs10999776的多态性分布

所有DNA标本均经AllGlo^TM^探针成功分型，DNA测序结果与实时荧光PCR结果一致。如图 1所示：A和B为实时荧光PCR的S形曲线，其中A表示野生型探针发生反应，而突变型探针未反应，故该样本基因型为野生型纯合子CC；而B则表示突变型探针发生反应，而野生型探针未反应，样本为突变型纯合子TT。C和D则分别为与上述样本相对应的DNA测序结果，两者一致。对照组ENT3 rs10999776（C＞T）的基因型分布符合*Hardy*-*Weinberg*平衡（χ^2^=0.674, *P*=0.714）。对照组中ENT3 rs10999776（C＞T）3种基因型（CC、TC、TT）频率分别为58.4%、37.7%、3.9%，C和T等位基因频率分别为77.3%、22.7%；肺癌组中3种基因型（CC、TC、TT）频率分别为52.4%、41.6%和6.0%，C和T等位基因频率分别为73.2%和26.8%，两组之间基因型和等位基因分布均无统计学差异（χ^2^分别为2.488、2.290，P值分别为0.288、0.130），见表 2。

**1:**
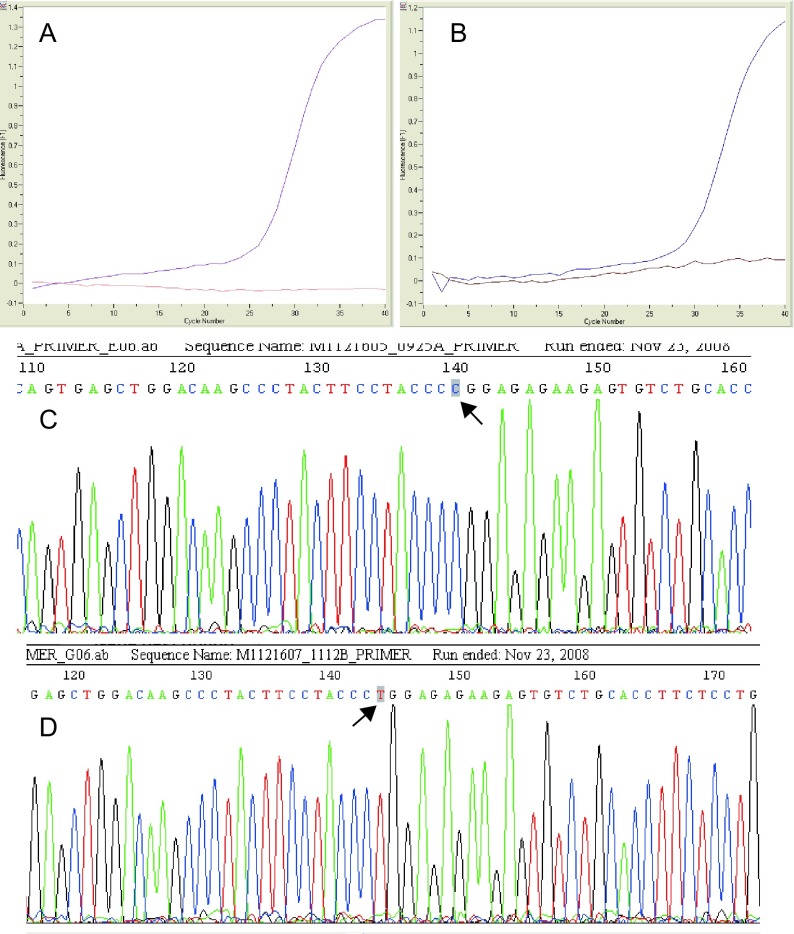
AllGlo^TM^探针检测的基因型结果与DNA测序验证 The genotype from AllGlo^TM^ probe assay and sequencing verification. A and B respectively denote real-time fluorescence curves of homozygous wild-type CC and homozygous mutation-type TT by AllGlo^TM^ probe assay; C and D show sequencing results corresponding with PCR. The arrows indicate allele.

**2:** 肺癌组和对照组基因型与等位基因分布 The distribution of genotypes and alleles in groups of lung cancer and control

Genotypes	Lung cancer (*n*=351)			Control (*n*=207)		*χ*^2^	*P*
	*n*	Constituent ratio (%)		*n*	Constituent ratio (%)		
Alleles						2.290	0.130
C	514	73.2		320	77.3		
T	188	26.8		94	22.7		
Genotypes						2.488	0.288
CC	184	52.4		121	58.4		
TC	146	41.6		78	37.7		
TT	21	6.0		8	3.9		

### 2.3 ENT3 rs10999776多态性与吸烟联合作用对肺癌风险性的影响

以携带野生基因型CC且非吸烟者为参照，累积吸烟量≥30包/年的野生基因型携带者患肺癌风险性增高，调整OR值为2.305（95%CI: 1.337-3.854, *P*=0.037）；而携带突变等位基因T的TC和TT基因型吸烟个体罹患肺癌的风险性更高，且≥30包/年者更甚，调整OR值分别为2.848（95%CI: 1.536-4.879, *P*=0.005）和3.076（95%CI: 2.308-6.741, *P*=0.001），见表 3。

**3:** ENT3 rs10999776多态位点基因型联合吸烟与肺癌风险性的关系 The relationship between the genotypes of rs10999776 combined with smoking and the risk of lung cancer

Genotypes	Smoking history (packs/year)	Lung cancer (*n*=351, %^a^)	Control (*n*=207, %^a^)	OR (95%CI)	*P*	OR^b^ (95%CI)	*P*
CC	Non-smokers	86 (24.5)	60 (29.0)	1.000		1.000	
	Smokers	98 (27.9)	61 (29.5)	1.121 (0.708-1.774)	0.626	0.985 (0.643-1.621)	0.702
	< 30	25 (25.5)	32 (52.5)	0.545 (0.294-1.012)	0.053	0.623 (0.375-1.233)	0.051
	> 30	73 (74.5)	29 (47.5)	1.756 (1.021-3.020)	0.041	2.305 (1.337-3.854)	0.037
TC+TT	Non-smokers	68 (19.4)	51 (24.6)	0.930 (0.570-1.519)	0.773	1.124 (0.604-1.623)	0.887
	Smokers	99 (28.2)	35(16.9)	1.973 (1.188-3.277)	0.008	2.848 (1.536-4.879)	0.005
	< 30	33 (33.3)	15(42.9)	1.535 (0.767-3.071)	0.224	1.637 (0.859-3.986)	0.159
	> 30	66 (66.7)	20(57.1)	2.302 (1.265-4.192)	0.006	3.076(2.308-6.741)	0.001
^a^: parentheses display constituent ratio; ^b^: OR adjusted by age and gender.							

### 2.4 ENT3 rs10999776多态性与吸烟联合作用对肺癌不同病理类型的影响

以携带野生基因型CC且非吸烟者为参照，发现3种基因型的吸烟个体罹患肺鳞癌的风险性均显著增高，但携带突变等位基因T的吸烟个体其风险性要高于野生型纯合子吸烟个体，OR值分别为6.066（95%CI: 2.884-12.758, *P*＜0.001）和2.573（95%CI: 1.238-5.348, *P*=0.01）。在野生型纯合子和携带突变等位基因T的个体中，吸烟≥30包/年者罹患肺鳞癌的风险性均更高，OR值分别为4.456（95%CI: 2.016-9.850, *P*＜0.001）和8.077（95%CI: 3.581-18.215, *P*＜0.001）；而三种基因型联合吸烟对肺腺癌的风险性并无明显影响（表 4）。

**4:** ENT3 rs10999776多态位点基因型联合吸烟与肺癌不同病理类型的关系 The relationship between the genotypes of rs10999776 combined with smoking and the pathologic types of lung cancer

Genotypes	Smoking history (packs/year)	AC (*n*=192)	SCC (*n*=100)	Control (*n*=207)	OR (95%CI)^a^	*P*^a^	OR (95%CI)^b^	*P*^b^
CC	Non-smokers	58	13	60	1.000		1.000	
	Smokers	46	34	61	0.780 (0.461-1.320)	0.355	2.573 (1.238-5.348)	0.010
	< 30	18	6	32	0.582 (0.295-1.150)	0.117	0.865 (0.300-2.493)	0.789
	> 30	28	28	29	0.999 (0.530-1.880)	0.997	4.456 (2.016-9.850)	0.000
TC+TT	Non-smokers	51	7	51	1.034 (0.609-1.758)	0.900	0.633 (0.235-1.708)	0.364
	Smokers	37	46	35	1.094 (0.608-1.966)	0.765	6.066 (2.884-12.758)	< 0.001
	< 30	16	11	15	1.103 (0.500-2.435)	0.807	3.385 (1.267-9.039)	0.012
	> 30	21	35	20	1.086 (0.534-2.211)	0.820	8.077 (3.581-18.215)	< 0.001
^a^: The comparison of the groups of lung adenocarcinoma (AC) and control; ^b^: The comparison of the groups of lung squamous cell carcinoma (SCC) and control.								

## 3 讨论

SNP作为人类最常见的DNA遗传变异，最早只被作为一种基因组作图的遗传标记，随着研究的不断深入，人们发现很多基因的SNP能够影响基因功能，与疾病易感性、药物疗效等相关，而日益引起研究者们的重视^[5]^。目前已经发现许多基因的SNP与肺癌易感性相关，如*XRCC1*、*SULT1A1*等^[6, 7]^。

核苷作为体内重要的低分子化合物，具有重要的生物学功能，如内源性核苷参与调节血液流动、糖代谢等重要生理功能。而核苷在细胞内的浓度将影响其功能的发挥。核苷的代谢和运输是影响核苷浓度水平的关键因素。而负责核苷跨膜转运的核苷转运蛋白十分重要。哺乳动物细胞主要有两大类核苷转运系统：平衡型转运系统（the equilibrative transport system, ENT）和集中型转运系统（the concentrative transport system, CNT）^[8, 9]^。前者是根据细胞内外核苷的浓度梯度进行跨膜运输，有4个家族成员ENT1-4，分布广泛；后者则是钠离子偶联性的而与浓度梯度无关，有6个家族成员。目前，对ENT1的研究较多，Sjoblom等^[3]^认为ENT1的遗传突变与结直肠癌的易感性有关，还有一些研究^[10, 11]^是集中于ENT1多态性或表达水平对核苷类似物抗肿瘤药如2, 2-二氟脱氧胞嘧啶核苷等疗效的影响。而对ENT3的报道并不多。Yamamoto等^[4]^分析了219个基因的5 438个SNPs位点，发现位于ENT3基因第二内含子上的多态位点rs10999776与晚期肺癌一线化疗的预后有关。

本研究结果显示，ENT3 rs10999776（C＞T）的CC、TC、TT基因型分布符合*Hardy*-*Weinberg*平衡（χ^2^=0.674, *P*=0.714）。肺癌组和对照组之间的基因型和等位基因分布无统计学差异（χ^2^=2.488, *P*=0.288; χ^2^=2.290, *P*=0.130）。

吸烟是肺癌的主要致癌因素，本研究将基因型与吸烟联合分析，结果显示累积吸烟量≥30包/年的野生型纯合子个体罹患肺癌的风险性增加；而携带突变等位基因T的吸烟个体患肺癌的风险性更高，调整OR值分别为2.305（95%CI: 1.337-3.854, *P*=0.037）和2.848（95%CI: 1.536-4.879, *P*=0.005）。累积吸烟量≥30包/年的T等位基因携带者其风险性又进一步提高，OR值为3.076（95%CI: 2.308-6.741, *P*=0.001）。这些结果提示突变等位基因T联合吸烟对肺癌风险性具有一定贡献作用。

肺癌分为不同的病理类型，它们的病因、组织发生、生物学特性等均各不相同。而在对该位点3种基因型联合吸烟与肺癌病理类型的分析发现：与非吸烟的野生型纯合子个体比较，该位点3种基因型的吸烟个体均有较高的罹患肺鳞癌的风险。而携带突变等位基因T的吸烟个体要比野生型吸烟个体的风险性更高，患病风险分别增加了6倍和2倍。累积吸烟量≥30包/年的个体，CC纯合子和TC+TT携带者的患病风险则分别增加了4倍和8倍。而基因型联合吸烟对肺腺癌无明显影响。这些结果提示ENT3 rs10999776的3种基因型和吸烟联合作用可能参与调节肺鳞癌的易感性。虽然该多态位点位于内含子，并未引起氨基酸残基的改变，但并不能排除它可能影响剪切位点的活性从而影响基因转录，或者该位点与其它功能位点存在连锁不平衡。这些相关机制需要进一步的深入研究加以确认。由于ENT3是一种核苷运载体，其功能主要是调节细胞内外的核苷或核苷类似物的浓度，进而影响到细胞的功能。但是它与吸烟之间的关系我们推测该基因可能与其它烟草代谢相关基因存在某些相互作用。这也需要进一步的研究加以证实。

本研究首次对核苷转运蛋白基因*ENT3*的单核苷酸多态性与中国人群肺癌易感性的关系进行了初步探讨。ENT3 rs10999776遗传多态性联合吸烟可能对肺癌易感性产生影响，并且可能与吸烟剂量相关。但是，由于肿瘤的复杂性，任何单一基因或多态位点的变化均不能完全说明和解决问题，该遗传多态性对肺癌易感性的影响只是对基因遗传多态性与肺癌易感性的一个补充，需要挖掘更多的肺癌易感性相关基因和多态位点以及它们与环境暴露的相互作用，才能更有效地说明问题。
